# Spatial analysis of digital economy and its driving factors: A case study of the Yangtze River Delta City Cluster in China

**DOI:** 10.1371/journal.pone.0300443

**Published:** 2024-05-29

**Authors:** Haidong Zhong, Bifeng Wang, Shaozhong Zhang

**Affiliations:** 1 School of Economics and Management, Ningbo University of Technology, Ningbo, Zhejiang, China; 2 College of Information and Intelligence Engineering, Zhejiang Wanli University, Ningbo, China; East China Normal University, CHINA

## Abstract

The digital economy (DE) has become a major breakthrough in promoting industrial upgrading and an important engine for high-quality economic growth. However, most studies have neglected the important driving effect of regional economic and social (RES) development on DE. In this paper, we discuss the mechanism of RES development promoting the development of DE, and establish a demand-driven regional DE development model to express the general idea. With the help of spatial analysis toolbox in ArcGIS software, the spatial development characteristics of DE in the Yangtze River Delta City Cluster (YRDCC) is explored. We find the imbalance of spatial development is very significant in YRDCC, no matter at the provincial level or city level. Quantitative analysis reveals that less than 1% likelihood that the imbalanced or clustered pattern of DE development in YRDCC could be the result of random chance. Geographically weighted regression (GWR) analysis with publicly available dataset of YRDCC indicates RES development significantly promotes the development of DE.

## 1. Introduction

With the wide application of information technologies, such as the Internet, wireless communication and artificial intelligence, the importance of DE is being promoted to a very high level. Early in 1998, the U.S. Department of Commerce released a report called *the Emerging Digital Economy* [1]. Subsequently, the U. S., Japan, Singapore, France and many other countries around the world have introduced DE strategies. For example, in 2016, the United States released the National Artificial Intelligence Research and Development Strategic Plan, and Germany released the Digital Strategy 2025 [2]. In 2022, the total DE of the top five economies in the world was 31 trillion US dollars, account for 58% of Gross Domestic Product (GDP), and an increase of about 11 percentage points over 2016 [3]. At present, DE has become an important strategic direction for many countries and a key force driving global economic growth.

In 2017, the Chinese government officially put forward the concept of DE in the Government Work Report, and then it booms in the nation. In recent years, the government departments at all levels in China have issued many policies and regulations to support the development of DE, and basically formed a sound policy system that combines the long-term planning goals of DE with detailed local promotion measures. According the report released by the China Communications Academy in 2023, the total amount of DE in China was 50.2 trillion yuan in the former year, and the nominal growth rate year-on-year reached up to 10.3%, which was significantly higher than GDP growth rate for 11 consecutive years [4]. Meanwhile, the proportion of DE in GDP is equivalent to the proportion of secondary industry in the national economy, reaching 41.5%. All these indicate the significant role of DE in driving China’s economic development.

Through the deep integration of DE and real economy with digital technology, most scholars believe that the overall economy and society can achieve continuous improvement and development in cost, efficiency, quality and scope [5, 6]. There are lots of research results on the positive role of the DE in boosting the mobility of production factors, reducing transaction costs, stimulating consumption potential, promoting innovation, and RES development [7, 8]. However, there are still the following two aspects need to be further explored: first, most scholars have paid much attention to the driving effect of DE on RES development, but little effort has been placed on the impact of the latter on the former, and second, YRDCC is a leading region in the development of DE in China, however, few studies have focused on spatial pattern and its driving factors of DE development in the region.

In this paper, we discuss the internal logic that RES development promoting the development of DE, and establish a conceptual model to represent the whole idea in detail. Possible contribution of the paper lies in the following three aspects:

1. The mechanism of RES development promoting the development of DE is explored, and a demand-driven regional DE development model is proposed.
2. Based on publicly available and authoritative DE development evaluation indicators, the spatial pattern of DE development in YRDCC is analyzed intensively.
3. With the help of GWR analysis tools in ArcGIS software, driving factors of DE development in YRDCC is investigated quantitatively, and the promoting effect of RES development on DE is verified accordingly.

The rest of the paper is structured as follows: In the following chapter, prior literature, such as the concept, connotation and evaluation index system of DE, spatial analysis of DE, and factors affecting the development of DE are reviewed. In Chapter 3, we establish and explain the theoretical model of RES development promoting the development of DE. Chapter 4 describes study area, data sources and research methods in detail. In Chapter 5, we investigate the spatial pattern of DE development in YRDCC. In Chapter 6, we take YRDCC as an example to conduct the empirical study on the relationship between RES and DE development with spatial analysis. Finally, we conclude the paper with shortcomings and future work in the last chapter.

## 2. Literature review

### 2.1. Definition and connotation of DE

The statement DE first appears in a book named *Digital Economy* [9]. In the book, DE is described as an all-encompassing lifestyle created by the advances in human communication, computing (computers, software, services), and content (publishing, entertainment, and information providers) in the Internet age. Up to now, there is no consensus among academics on the definition of DE. Some scholars define it as an economic form that uses modern information technology to digitize business processes and business activities in various industries [10]. And other scholars believe that DE is a new economic format based on network communication, artificial intelligence, big data, etc., taking data as the core production factor to realizing the collaboration and integration between data and traditional production factors [8, 11]. Currently, the most widely accepted definition of DE appears in the set out of the *G20 Digital Economy Development and Cooperation Initiative*: *“*A series of economic activities that use digital knowledge and information as key production factors, modern information networks as an important carrier, and the effective use of information and communication technologies as an important driving force for improving efficiency and optimizing economic structure” [12].

Compared with the traditional economy, DE is regarded as a new economic form which is based on information technology. With the help of different information technologies, various resource elements can flow freely and quickly, market players can restructure their organizational models, and a large number of market players can accelerate their integration to achieve cross-border development [11]. DE has undergone comprehensive changes in production factors, production relations and productivity. The changes that DE is likely to bring lies in the following three aspects [10, 13]: (1) Digitalization. DE excavates social and economic activities through information systems, the Internet of Things (IoT) sensing, machine vision and other digital ways to form data, information and knowledge that can be recorded, stored and interactive. In this process, data becomes a new means of production and a key factor of production. (2) Networking. The collected data, information and knowledge can flow freely, seamlessly and comprehensively through the Internet, IoT and other network carriers. This may greatly change the traditional production relations, and (3) Intelligence. Realize automatic and intelligent data processing with IT systems, big data, cloud computing, artificial intelligence and other advanced information and communication technologies. This can make the efficiency of social and economic activities improve rapidly, and the social productivity increase exponentially.

### 2.2. Development evaluation of DE

At present, scholars have not reached a consensus, to evaluate the development level of DE, and many international organizations, government statistical agencies and scholars have proposed many different measurement methods. However, most of them measure the scale of DE by calculating its added value and construct digital economy index (DEI) based on other comprehensive evaluation indicators. Typically, the European Union published the Digital Economy and Society Index (DESI) annually to evaluate DE development for countries within the EU since 2014. DESI is calculated by thirty-one secondary indicators within five main aspects, such as broadband access, Internet application and so on [14]. The United States Department of Commerce Digital Economy Measurement Framework that includes the degree of digitalization in various sectors of the economy, the impact of digitalization in economic activities and output, the comprehensive influence of economic and social development related factors, and the monitoring of emerging digitalization areas [15]. The Organization for Economic Cooperation and Development DE measurement indicator, which includes 38 indicators with international comparability, such as broadband penetration, ICT equipment and applications [16].

In China, most of the DE development evaluations are issued by well-known enterprises or scientific research institutes. They usually refer to DE index released by China Academy of Information and Communications Technology (http://www.caict.ac.cn/) and the "Internet +" DE index published by Tencent Group (https://www.tencent.com/en-us/index.html) [17]. For example, it is the case for the Global Digital Economy Competitiveness Index released by Shanghai Social Science Development Research (https://english.sass.org.cn/), the China Central City Digital Economy Index (CCCDEI) released by H3C Group (http://www.h3c.com/), and the China Digital Economy Index (CDEI) released the Caixin Insight Group (https://www.caixinglobal.com/company-info/caixin-insight-group-172.html) [18]. These kind of DE development evaluations usually utilize a lot of indicators that are not publically available or difficult to obtain. Also, there still many other published literatures evaluate the development level of the DE just based on publically available panel data. For example, DE evaluation indexes proposed by Ting, C. [19], Shuofeng, G. [20] and Bing⁃jie, S. [21]. However, compared with research institutions and government departments, the coverage of the DE evaluation index system established by scholars in academic papers is not so perfect. Data availability limitations may be one of the biggest challenges.

### 2.3 Spatial characteristics of DE development in China

In recent years, research on DE has become increasingly mature, more and more scholars pay attention to the spatial characteristics of DE development in China. With the extension of the connotation of DE, the studies are also expanding. At the regional level, Liu C., et al. analyzed the regional differences and dynamic evolution process of DE development through methods such as Kernel density and Dagum Gini coefficient [22]. They found that there were significant differences within and between the five major urban agglomerations, and there were gradient and multipolar development trends, showing an overall uneven distribution in China. At the provincial level, Wang B., et al. used interpolation simulation, Zipf order scale method, and geographic detector methods to analyze the spatial differentiation characteristics and influencing factors of DE in China. They found significant DE development differences exist in the eastern, central, western, and northeastern areas of the nation [23]; Zhang X. and Wu T. studied the spatial pattern of DE development in China with entropy and natural breakpoint methods. They found the development level of DE in the eastern area of China is much higher than the western area, and the unbalanced situation in provincial scale is also very significant [24]. At the municipal level, Zhong Y. and Mao W. explored the spatial distribution characteristics of DE development in China with spatial econometric models, and found there were significant differences between cities located in upstream and downstream of the Yangtze River Economic Belt [25]; Tian J., et al. analyzed the spatial differentiation pattern of DE development in Northeast China with multiple methods, such as the Thiel index and geographic detectors, and found that the overall development level of DE in Northeast China was relatively low, and the polarization of internal urban development was significant [26].

### 2.4. Relationship between RES and DE development

With the emergence of the digital wave in recent years, both academia and industry have realized that the rapid development of the DE has not only brought new growth points and driving forces to the global economy, but also played an increasingly important role in RES development [27]. A large number of studies have analyzed the positive effects of DE on RES development. Most of them believe that the development of DE can accelerate the cross-border integration of the local economy and enhance the overall competitiveness of the regional economy [28, 29]. First of all, the development of information technology makes traditional industries more efficient, intelligent and automated, and promotes the upgrading and transformation of regional economies. Secondly, the development of DE is conducive to the advancement of urbanization process, and the popularization of digital technology and e-commerce can accelerate the speed of urbanization and promote the rapid development of urban economy and society. Finally, the development of DE can promote the development of education, technology and other industries, and improving the population quality and technical ability of the entire region [30]. In China, the development of DE is not only related to the economic and social development of the nation, but also an important means to promote the construction of a modern economic system and build a new national competitive advantage [28].

Most studies start from spatial perspective to find the factors that influence the spatial pattern of DE. The investigations reveal that information technology foundation, geographical region, economic level, and human capital have a significant impact on the spatial pattern and evolution of DE [20]. Over time, the impact of information technology foundation and human capital has become increasingly important, while the impact of geographical regions on DE has gradually weakened [31–33]. Still some other studies have shown that economic growth, foreign investment dependence, industrial structure optimization, government behavior, urbanization, and human capital have significant impact on regional DE development [11, 34, 35]. Overall, there are various indicators affecting the development of DE, however, relatively a few of them come from the perspective RES development.

## 3 The mechanism of RES development promoting the development of DE

There is very close relationship between RES development and the development of DE. Generally, the higher the level of RES development and the more efficient the governance of a region, the more perfect its digital infrastructure construction, the stronger policy support, and the higher the level of DE development. On the contrary, the lower the level of RES development and the less efficient governance in a region, the lower the level of information industry development and the fewer DE activities [36].

**Fig 1:**
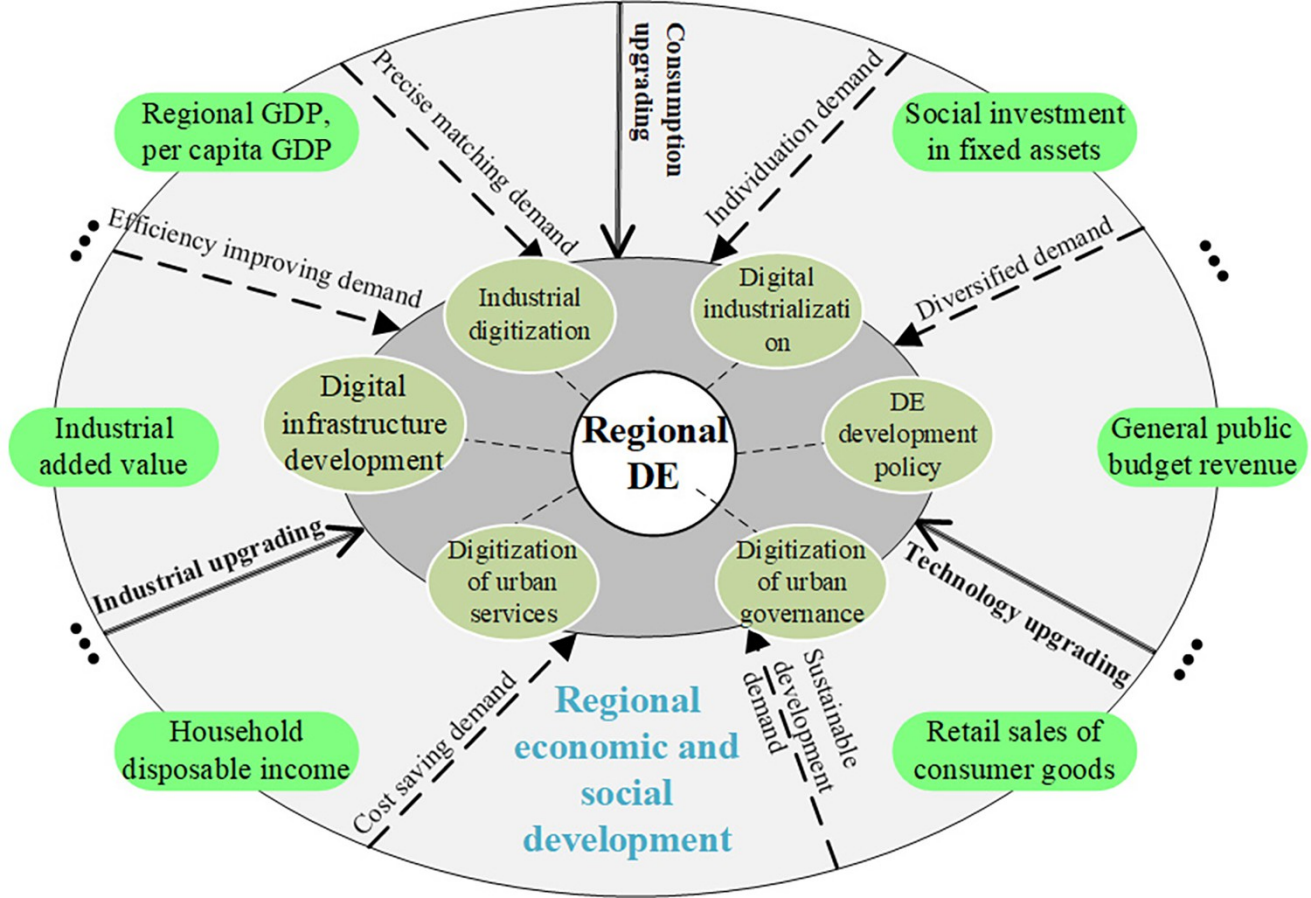
Demand-driven regional DE development model.

First of all, the development of RES directly contributes to consumption upgrading, industrial upgrading and technology upgrading [37]. Secondly, with the development of RES, people’s demands, such as efficiency improvement, individuation, precise matching, sustainable development, efficiency improving and cost saving will continue to emerge and upgrade [38]. And these will ultimately promote the development of DE. Following this approach, we establish a RES development promoting the development of DE model (as shown in Fig 1) based on the demand-driven theory [39, 40]. In this model, we select commonly used indicators, such as retail sales of consumer goods, GDP, per capita GDP, industrial added value, household disposable income of urban residents, general public budget revenue, social investment in fixed assets, etc. to represent the development level of RES quantitatively. Meanwhile, industrial digitization, digital industrialization, DE development policy, digitization of urban governance, digitization of urban services, digital infrastructure development, etc. are selected as indicators to represent the level of DE development.

### 3.1 RES development promote consumption upgrading

As China moves toward high-quality development in recent years, the contribution of consumption to economic growth is increasing day by day [27]. However, most researches neglect the promoting effect of RES development on consumption upgrading. The promoting effect falls into the following two aspects: Firstly, RES development inevitably leads to an increase in product output and labor services, leading to the emergence of new industrial sectors and new commodities. This will expand consumers’ demand and the scope of consumption. Secondly, with the growth of RES and the increase of the GDP, per capita disposable income, household disposable income of urban residents, etc., people’s living standards and purchasing power will increase continuously, leading to an increase in the level of consumer demand. In the past, the commodities that may have been consumed were of relatively poor quality and low grade. Nevertheless, with the development of RES, people’s consumption of commodities will upgrade, which is manifested in higher demands for the commodities quality and optimization of the consumption structure.

### 3.2 RES development promote technology upgrading

It is generally believed that scientific and technological progress plays a very important role in RES development [41]. But the role of the latter in boosting the former has been underestimated for a long time. Firstly, RES development can bring a great many high-leveled skilled labor to gather and improve the overall regional human resource level. And the level of human capital somewhat determines the optimal allocation ability of labor force among industries and the type of technological progress. A lot of empirical evidence shows that different types of human capital have different effects on technological upgrading [27, 42]. The more workers in a region with high labor proficiency, high technical and education levels, the more significant the effect of human capital on technological progress. Secondly, RES development provides capital for technological upgrading. The more developed a country’s economy, the more financial resources it can afford to support and promote technological progress. The experience of scientific and technological development of countries around the world shows that the regional distribution of research and development funds is closely related to a country’s level of economic and social development. In the early 1970s, developed countries, e.g., the UK, Germany, the former Soviet Union, former West Germany, Japan, the US, and France accounted for 85% of the world’s research and development expenditure, while developing countries in Africa and other regions only accounted for 3% [43].

### 3.3 RES development promote industrial upgrading

RES development provides the basic guarantee for the technological innovation of regional enterprises, the expansion of industrial scale, the adjustment of industrial structure and the necessary material basis for industrial upgrading. Firstly, regional economic development can prompt the government to enhance efficiency and introduce more policies conducive to enterprise innovation and upgrading, such as tax incentives and financial subsidies. This provides a good policy environment for the transformation and upgrading of enterprises. Secondly, economic growth triggers the promotion and improvement of regional education, medical care and infrastructure construction, and attract more high-end talents to gather. This provides an important human resource guarantee for industrial upgrading. Thirdly, with the development of economy, consumers’ demand for products and services continues to increase, and enterprises must improve product quality, reduce costs and optimize services through technological innovation, management innovation and other means to meet market demand. This provides a direct impetus for the transformation and upgrading of enterprises. Finally, RES development leads to the formation of a gradient development pattern between central cities and surrounding cities in the process of industrial transfer and docking, which promotes the optimal allocation of resources and the upgrading of industries [44]. The faster the regional economy develops, the faster the speed of technological progress and innovation, providing more opportunities for enterprises to transform and upgrade.

## 4. Study area, data sources and research methods

### 4.1. Study area

**Fig 2:**
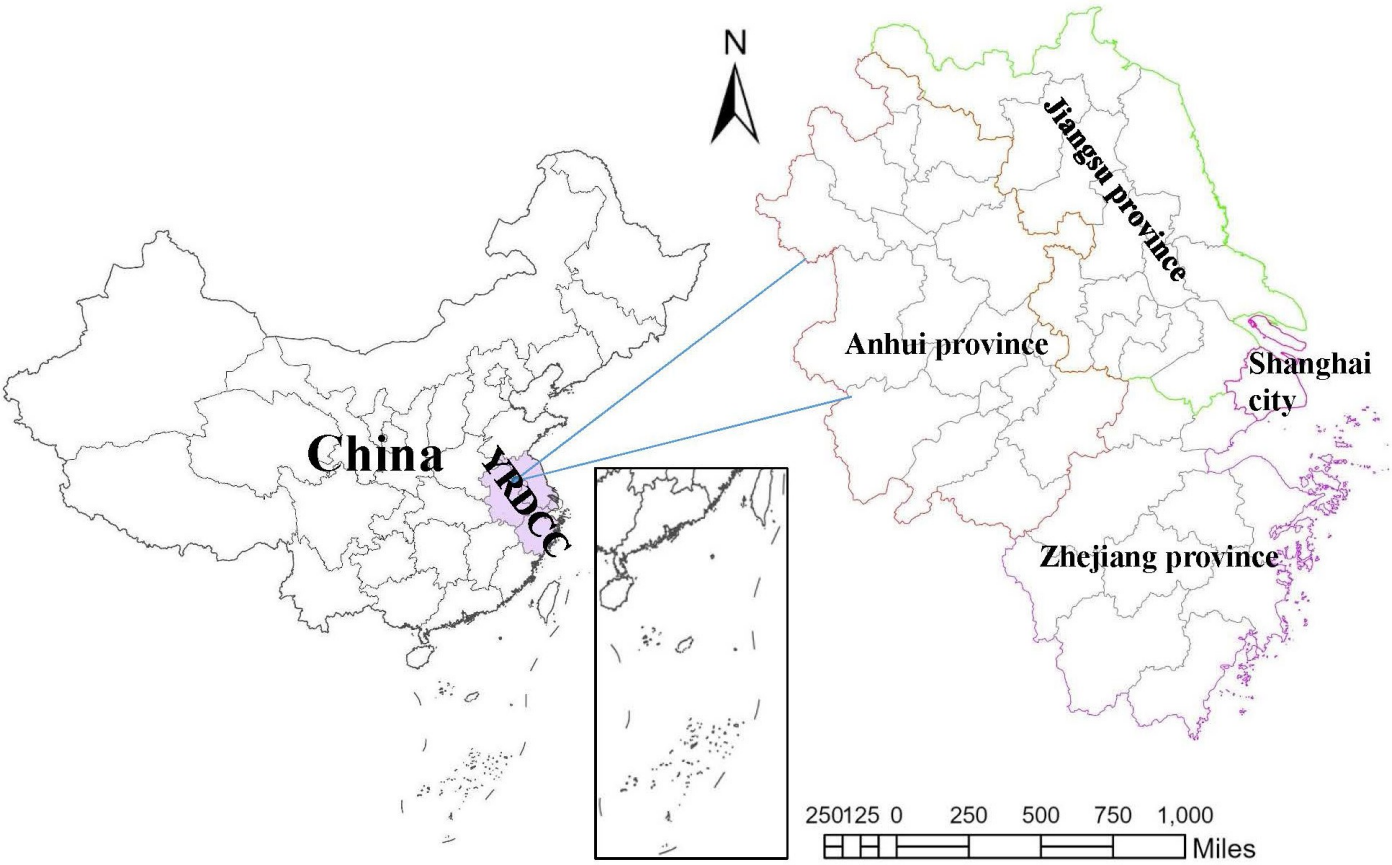
The location of YRDCC.

YRDCC lies at the heart of China’s eastern coast, where the Yangtze and Huai Rivers meet at the mouth of the sea (as shown in Fig 2). It is one of the six major city clusters, an important birthplace of China’s industry and commerce, famous manufacturing center in the world, and one of the regions with the most dynamic economy and the greatest growth potential in China [45]. YRDCC contains a total of 26 cities in three provinces and one city, covering an area of 21,700 square kilometers. Meanwhile, YRDCC is one of the regions in China with the best urbanization foundation, and rich natural and cultural resources. It is one of the important centers of China’s economic, cultural and social development, and a pioneering demonstration zone for China’s modernization and opening-up. YRDCC is also deemed as one of the main portals of Western culture into China, and the most international and modern regions in China [46].

YRDCC is the most economically developed and urban agglomeration area in China. The overall economy of the region is large, with many strong enterprises and industrial clusters. There is also a relatively obvious hierarchical structure and functional division of labor among cities, such as Shanghai’s status as an international financial center and scientific and technological innovation center as a core city, Nanjing, Suzhou and other cities’ manufacturing and scientific and technological innovation capabilities, and Hangzhou’s DE and high-tech industrial advantages [47]. The economic hinterland of YRDCC is vast, with modern river and sea port clusters and airport clusters. The highway network is very sound, and the density of railway transportation trunk lines is leading in the country. In addition, YRDCC has about 1/4 of the country’s "double first-class" construction universities, and a large number of high-level scientific and technological talents [48].

### 4.2. Data sources

The dataset used in the research falls into the following four categories, their names and sources are detailed as follows:

1. Basic geographic information data of YRDCC and China. This data is downloaded from GaryBikini (GaryBikini/ChinaAdminDivisonSHP:v23.01.04, 2023, DOI: 10.5281/zenodo.7503181). In this paper national, provincial, and municipal boundaries of the country are used to show the location of YRDCC (as shown in Fig 2).
2. RES development related indicators for the cities in YRDCC. These indicators include permanent population, GDP, total retail sales of consumer goods, general budget revenue, per capita disposable income of urban residents, per capita GDP, etc. The data is collected from Shanghai Statistical Yearbook (https://tjj.sh.gov.cn/tjnj/index.html), Zhejiang Statistical Yearbook (https://tjj.zj.gov.cn/col/col1525563/index.html), Jiangsu Statistical Yearbook (http://tj.jiangsu.gov.cn/col/col89815/index.html), and Anhui Statistical Yearbook (http://tjj.ah.gov.cn/ssah/qwfbjd/tjnj/index.html).
3. DE development index of cities in YRDCC. Based on the comprehensive considerations of authoritative and data availability, the paper uses CCCDEI, released by H3C Group (http://www.h3c.com/cn/d_202011/1355635_30008_1.htm), to measure the level of DE development of cities in YRDCC. CCCDEI consists of four primary indicators: data and information infrastructure, urban services, urban governance, and industrial integration; twelve secondary indicators, including information infrastructure, data infrastructure, and operational infrastructure, and so on; forty six tertiary indicators, including the penetration rate of fixed network broadband applications, the number of policies covering people’s livelihoods, the comprehensive index of industrial integration, etc. [49].

### 4.3. Research methods

A large number of existing studies have shown that geographical analysis has unique advantages in ecology, soil science, regional economics and other fields [50, 51]. In this paper, global spatial autocorrelation (GSA) analysis method is used to explore the general regularity of spatial distribution of DE development in YRDCC, and local spatial autocorrelation (LSA) analysis approach is utilized to conduct an in-depth study. In addition, GWR analysis is utilized to find out the influencing factors of DE development in the region.

#### 4.3.1 GSA analysis

GSA focuses on analyzing the spatial distribution state and pattern of attribute values of spatial objects in the whole region, and the commonly used statistics is Moran’s I. It can reflect the similarity degree of the attribute values of units in adjacent or adjacent regions of space, and deemed as one of the most widely used statistics at present. There are two hypothesis testing methods for Moran’s I statistics: random distribution and approximate normal distribution [52].

GSA analysis has been implemented in ArcGIS software. By using spatial analysis toolbox of the software, Moran’s I Index value can be obtained easily. Meanwhile, there is another index, Z-score, is calculated accordingly to validate the effectiveness of the former. According to the value of Z-score, results of the spatial analysis can be interpreted in the following ways: (1) if Z-score value is greater than zero, spatial distribution of the result is clustered, and the result is confident (at the 0.05 confidence level) if Z-score value greater than 1.96; (2) if Z-score value is smaller than zero, the calculated result is discrete, and the result is also confident (at the 0.05 confidence level) if the Z-score value smaller than -1.96, and (3) the result is random if Z-score value within [-1.96, 1.96]. Moran’s I is calculated as follows [53].

$$I=\frac{n}{S_{0}}\frac{\sum_{i=1}^{n}\sum_{j=1}^{n}W_{i,j}Z_{i}Z_{j}}{\sum_{i=1}^{n}Z_{i}^{2}}$$ (1)

where *n* is the total number of locations, *Z*_*i*_ is the deviation between the observed value and the expected value at location *i*, and *Z*_*j*_ is the deviation between the observed value and the expected value at location *j*. *W*_*i*,*j*_ is the proximity relation values between locations *i* and *j*, and *S*_0_ is the sum of all proximity values.

#### 4.3.2 LSA analysis

The test premise of GSA is the hypothesis of regional homogeneity, but in reality, heterogeneity is more common. Additionally, the global optimal generally does not represent the local optimal. Similarly, the macro conclusions, cannot cover up some micro problems, and a high degree of global autocorrelation does not mean a high degree of local correlation. Therefore, we need a model that can explore and analyze spatial distribution on a more microscopic scale. LSA improves the Moran’s I model by breaking down the overall relationship to make each component can be calculated. Compared with GSA, LSA can better grasp the clustering and differentiation characteristics of local spatial elements by comparing the relationship between observed values and adjacent values, and the global [51]. LSA analysis can be used to test the cluster region, and verify the hot and cold spots where observations are clustered.

In ArcGIS software, LSA analysis is included in the spatial analysis toolbox as Anselin Local Moran’s I, which can be figured out according to Formula (2).

$${LMI}_{i}=\frac{n(x_{i}-\bar{x})\sum_{j=1}^{n}w_{ij}(x_{j}-\bar{x})}{\sum_{i=1}^{n}{\left(x_{i}-\bar{x}\right)}^{2}}$$ (2)

where *n* is the total number of locations, *w*_*ij*_ is the row normalized form of the spatial weight matrix at locations *i* and *j*, *x*_*i*_ is the observed value at location *i*, *x*_*j*_ is the observed value at location *j*, and $\bar{x}$ is the average value of *x*_*i*_ and *x*_*j*_. A positive *LMI*_*i*_ value indicates the property value at location *i* is similar to that of its neighbors, and the opposite is true when *LMI*_*i*_ is negative.

#### 4.3.3 GWR analysis

GWR is a kind of space-based statistical method, which consider the influence of each spatial unit according to the spatial coupling characteristics. In GWR, the regression parameters for a location are no longer obtained from observed value at the spot, but estimated with observations from neighboring data, and these variables vary with spatial locations [52]. Therefore, GWR can effectively analyze spatial correlations, identify spatial influences, find decision-making errors in time so as to adjust expected performance early, and make the analysis more accurate. GWR is also included in ArcGIS spatial analysis toolbox, and its calculation be represented by the following formula [53]. Following previous studies [54–56], we assumed that the given error term in Eq (3) is normalized at zero mean value and constant variance.

$$y_{i}=f_{0}(u_{i},v_{i})+\sum_{k=1}^{n}f_{k}(u_{i},v_{i})x_{i,k}+\varepsilon_{i},i\in [1,n]$$ (3)

where *u*_*i*_ and *v*_*i*_ are the geographical coordinates at location *i*, *f*_*k*_(*u*_*i*_, *v*_*i*_) is the *k*th regression parameter on the sampling location *i*. And *f*_*i*_ is a function of geographical location, which is obtained by the method of weight function in the estimation process.

## 5. Spatial pattern of DE development in YRDCC

To reveal the spatial distribution characteristics of DE development in YRDCC, the research conducts a spatial analysis with 2018–2021 CCCDEI of YRDCC in ArcGIS pro 2.5.

### 5.1 DE development evaluation in YRDCC

To achieve regional collaborative progress and narrow regional differences, the 14th Five Year Plan for National Economic and Social Development of the People’s Republic of China and the Outline of Long Range Goals for 2035 (https://www.ndrc.gov.cn/xxgk/zcfb/ghwb/202103/t20210323_1270124.html) clearly point out "improving the level of integrated development in the Yangtze River Delta". As one of the regions with the most reasonable urban hierarchical structure, close external cooperation, leading digital transformation and innovative ecological in China, the Yangtze River Delta region open its arms to welcomes new opportunities for integrated development. However, due to the imbalanced development of policy orientation and resource endowment, the development level of DE in YRDCC varies significantly [57].

**Fig 3:**
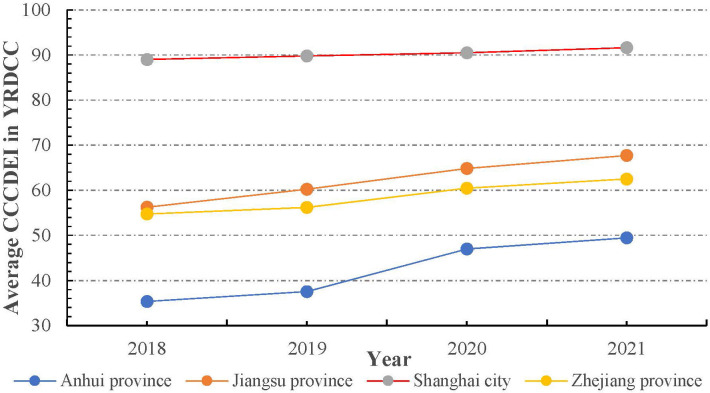
DE development of provinces in YRDCC from 2018 to 2021.

The CCCDEI does not provide provincial level DE development evaluation. We measure the DE development level of each province by averaging the CCCDEI of the cities under its jurisdiction, and the result is shown in Fig 3. Overall, DE grows in all four provinces (provincial-level city) between 2018 and 2021, and DE development level in Shanghai is the highest, while Anhui province is the lowest. However, evaluating from the growth rate of the average CCCDEI for 2018–2021, the situation is quite different. Specifically, the growth rates in Anhui province is highest 39.9%, while Zhejiang province is the lowest 14.2%, during the same period. In recent years, China has been implementing the Yangtze River Delta integration strategy. However, there is a lot of room for improvement of DE development in Zhejiang, Anhui and Jiangsu provinces, and it is still urgent to narrow the regional gap to avoid the widening of the "digital divide".

### 5.2 Spatiotemporal evolution of DE development in YRDCC

**Fig 4:**
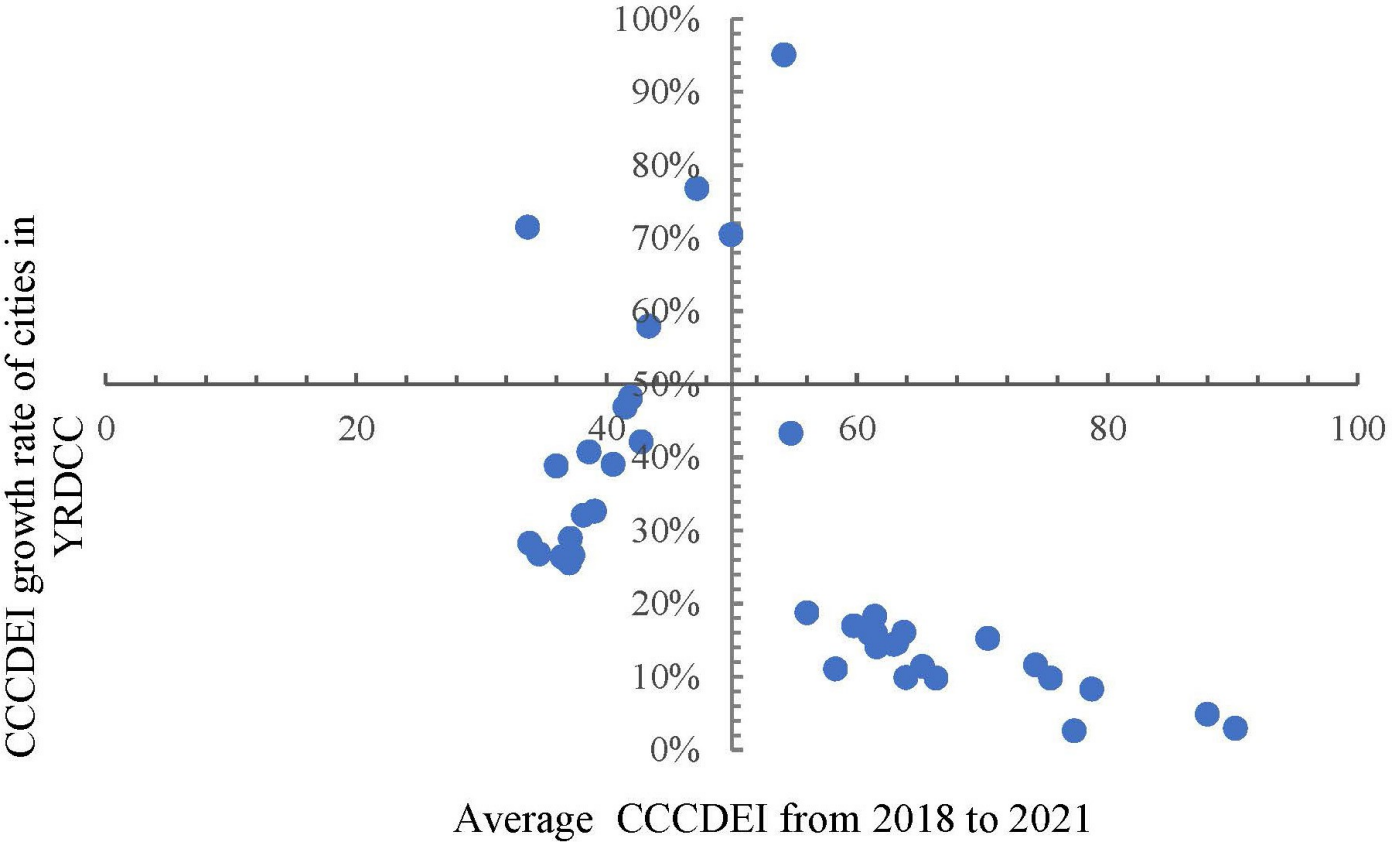
Average CCCDEI and its growth rate for cities in YRRDCC for 2018–2021.

As can be seen from Fig 4, the unbalanced and inadequate development of DE is a prominent problem, which is reflected in the low CCCDEI in most regions. The average value of Shanghai’s CCCDEI is nearly three times than that of Huaibei, Zhoushan, Quzhou and other cities. From 2018 to 2021, CCCDEI in four cities, Suqian, Huaibei, Tongling and Lianyungang, saw a growth rate of more than 70%. Although the four cities have large growth rates, the gap between them still significant.

From the city level perspective, cities with high CCCDEI growth rate are mostly concentrated in the east of the Yangtze River Delta and coastal areas, while cities with low CCCDEI average growth rate are mostly concentrated in the western region. All these imply that the difference of DE development in YRDCC is difficult to be closed in a short time, and there is an obvious "Matthew effect" [58].

**Fig 5:**
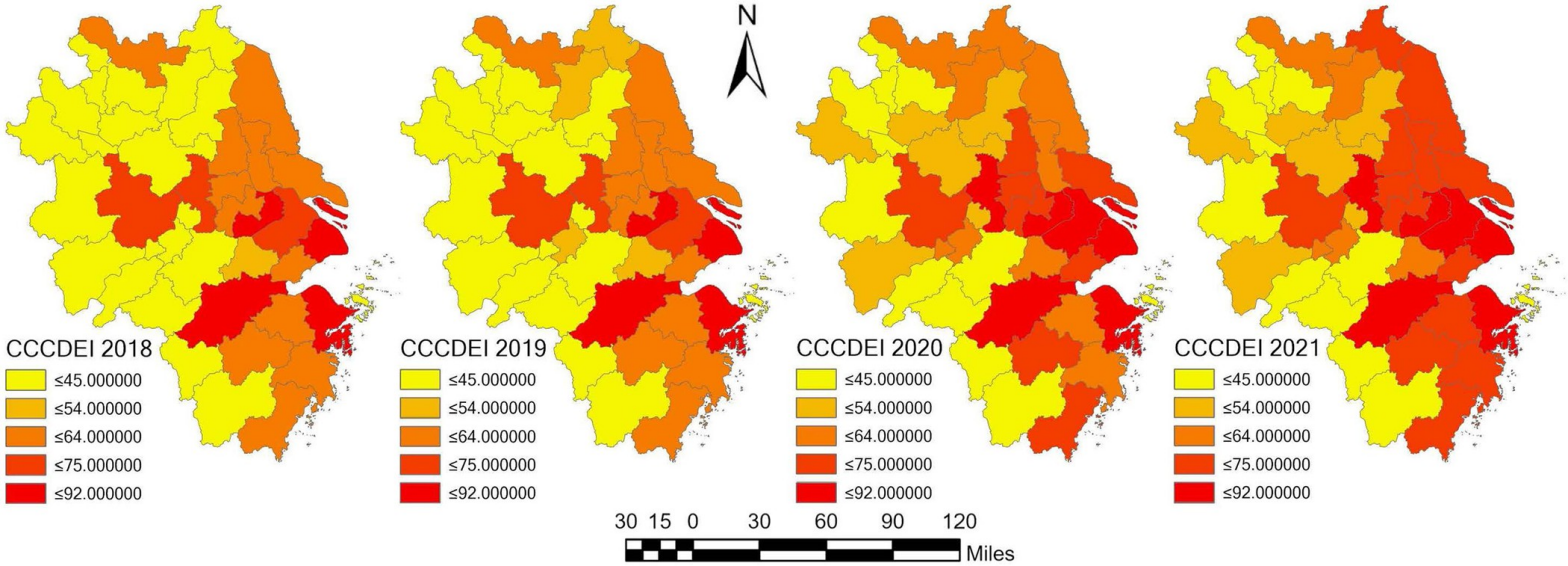
Spatial distribution of DE development in YRDCC from 2018 to 2021.

Fig 5 shows the spatial distribution of DE development in YRDCC displayed in graduated colors with the same color scheme and intervals. It indicates that the development level of DE in YRDCC has significant spatial heterogeneity from 2018 to 2021. On the whole, DE development level of cities in the east of YRDCC is relatively high, while in the west it is relatively low. Specifically, most cities under the jurisdiction of Anhui province have low DE development level. These cities include Bozhou city, Huaibei city, Xuancheng city, Fuyang city, Suzhou city, Chizhou city, Lu’an city, Huainan city, Huangshan city, Maanshan city, Chuzhou city, Bengbu city and Anqing city. Shanghai’s DE development has maintained at a relatively high level from 2018 to 2021, and this is also the case in most cities in Zhejiang province. However, there still many cities, such as Suzhou city, Huaibei city, Huainan city, Lishui city, Quanzhou city, Bozhou city, etc. have experienced a relative decline of DE development in the same period.

### 5.3 Spatial correlation of DE development in YRDCC

To check whether spatial autocorrelation exists in the development of DE in YRDCC, we conduct the Global Moran’s I analysis with the help spatial analysis toolbox in ArcGIS pro, and the results are shown in Table 1. The main parameters’ settings for the analysis are as follows: (1) Conceptualization of Spatial Relationships: Contiguity edges corners; (2) Distance method: Euclidean, and (3) Standardization: Row. It can be seen from Table 1 that the z-score values are greater than 2.58 in all years, while the p-value values less than 0.01. According to the spatial autocorrelation determination rules [59], there is a less than 1% likelihood that the clustered pattern of DE development in YRDCC could be the result of random chance.

**Table 1:** Spatial autocorrelation report of CCCDEI in YRDCC for 2018–2021.

	2018	2019	2020	2021
**Moran’s Index**	0.358784	0.327292	0.298252	0.303245
**Expected Index**	-0.025	-0.025	-0.025	-0.025
**Variance**	0.010089	0.010092	0.010062	0.010093
**z-score**	3.820825	3.506757	3.222529	3.267259
**p-value**	0.000133	0.001271	0.000454	0.001086

**Fig 6:**
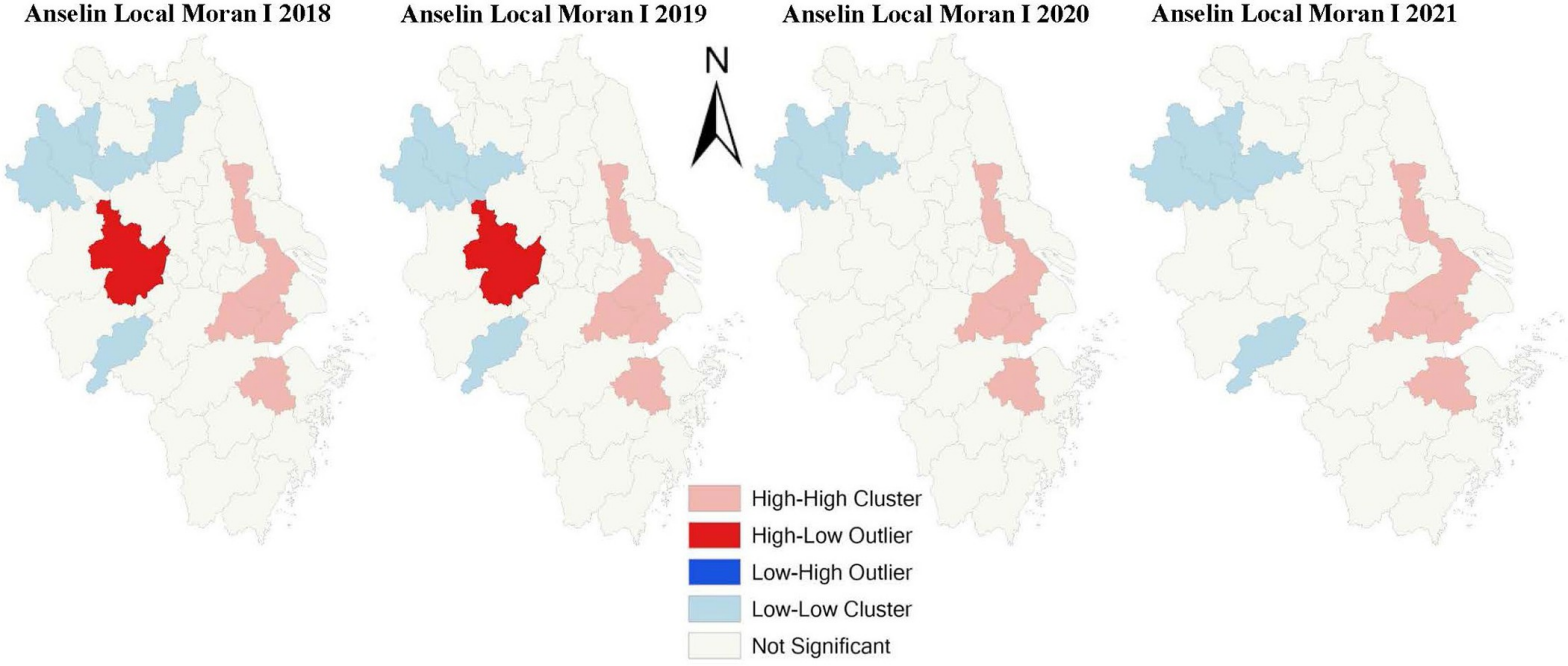
Anselin Local Moran I of CCCDEI in YRDCC.

Based on the overall clustered pattern of DE development conclusion of the Global Moran’s I analysis, we try to conduct the Anselin Local Moran I analysis to find more accurate and specific results. The main parameters are set the same as the Global Moran’s I analysis in ArcGIS software, and the results are shown in Fig 6. The results demonstrate that CCCDEI of cities in YRDCC presents a highly concentrated pattern in space. Specifically, there are two High-High clusters, where are DE well-developed hot places in the east of YDRCC from 2018 to 2021. One of the clusters is located in the northeast that contains four cities in Jiangsu province. They are Hangzhou city and Huzhou city in Zhejiang province, and Taizhou city and Suzhou city. The other cluster contains only one city, Shaoxing city in Zhejiang province.

In 2018, 2019 and 2021, there are two Low-Low clusters, where are DE development cold places, and the clusters contain almost the same cities in the three years. From the analysis result of 2021, one of the Low-Low clusters in the northwest contains four cities in Anhui province. They are Bozhou city, Huaibei city, Bengbu city, Huainan city, and Fuyang city. While the other Low-Low cluster contains only one city, Chizhou city of the same province. Additionally, the High-High and Low-Low cluster areas have changed little between 2018 and 2021. It is still necessary to improve the market mechanism, cooperation mechanism, mutual assistance mechanism and support mechanism to narrow DE development gap between cities in the Yangtze River Delta at present.

There are few High-Low cluster areas, including only Hefei city in 2018 and 2019, which shows the level of DE development in the city is high, but its neighbor cities are low. The phenomenon of "polarization" is easily generated in High-Low cluster region. However, in the following two years, the agglomeration characteristics of Hefei city is not significant, showing an overall good DE development tendency.

## 6 Driving factors of DE development in YRDCC

### 6.1 Construction of GWR analysis model

To verify the rationality of the proposed demand-driven regional DE development model and explore the driving factors for the spatial pattern of DE development in YRDCC, we select city-level CCCDEI and RES development related indicators to establish the following GWR model (as shown in Formula (4)). The analysis is completed with the help of GWR analysis toolkit in ArcGIS software. We set the model type to Continuous (Gaussian) and the local weighting scheme to Gaussian. The parameters in the model are explained in the Table 2.


$${CCCDEI}_{i}=f_{0}(u_{i},v_{i})+\sum_{k=1}^{n}f_{1}(u_{i},v_{i})x_{i,k}(GDP)+\sum_{k=1}^{n}f_{2}(u_{i},v_{i})x_{i,k}(TPROUDCTVALU)+\sum_{k=1}^{n}f_{3}(u_{i},v_{i})x_{i,k}(AVGGDP)+\sum_{k=1}^{n}f_{4}(u_{i},v_{i})x_{i,k}(BUDGETREV)+\sum_{k=1}^{n}f_{5}(u_{i},v_{i})x_{i,k}(URBANUSEABLE)+\sum_{k=1}^{n}f_{6}(u_{i},v_{i})x_{i,k}(SOCIALSALES)+\varepsilon_{i}$$ (4)

**Table 2:** GWR model parameters description.

OLS model parameter	RES development related indicator
*GDP*	Gross domestic product
TPROUDCTVALU	Added value of the tertiary industry
AVGGDP	Per capita gross regional product
BUDGETREV	General budget revenue
URBANUSEABLE	Household disposable income of urban residents
SOCIALSALES	Total retail sales of consumer goods

**Fig 7:**
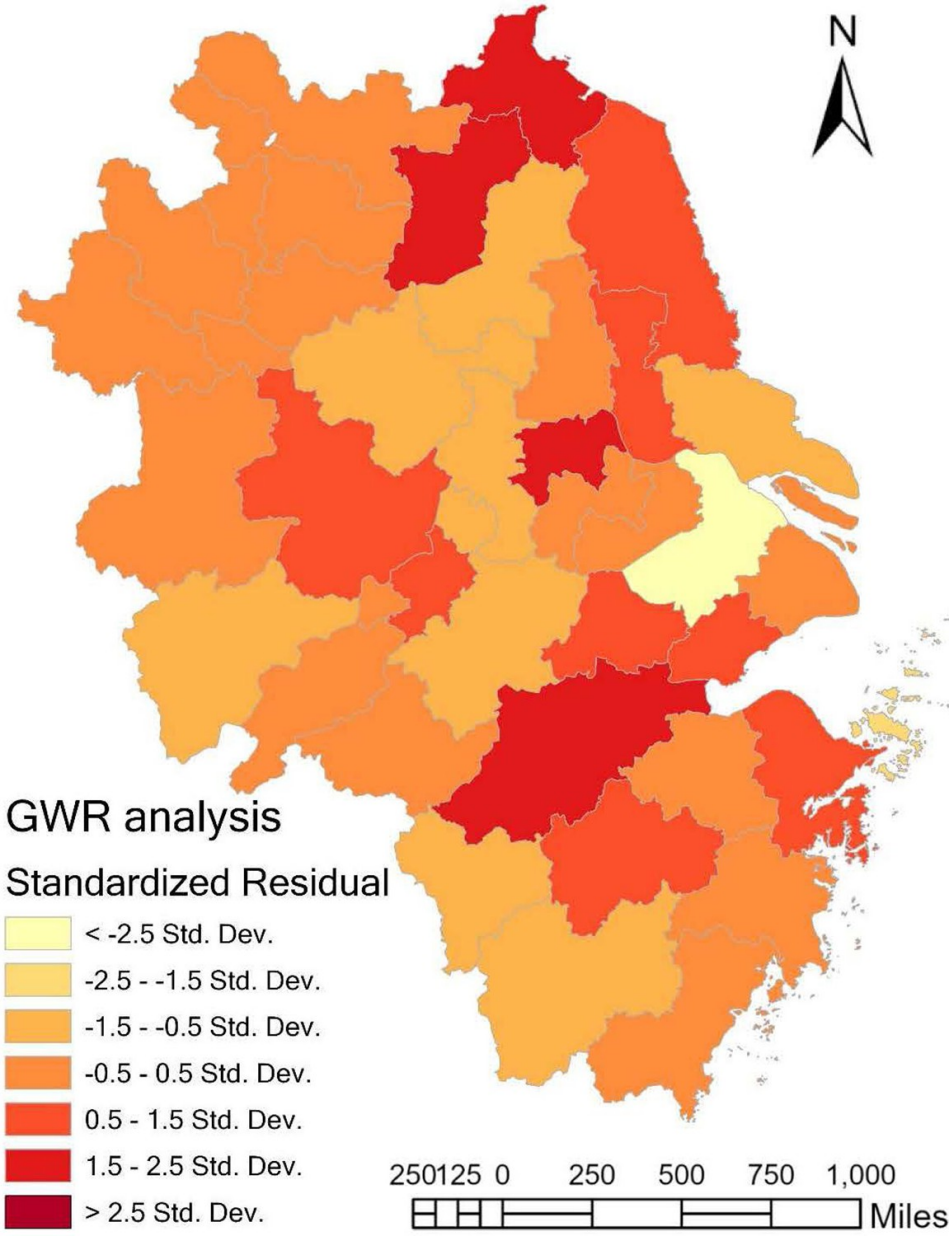
Spatial distribution of residual in GWR analysis.

Due to the impact of the COVID-19, from January 2020 to the end of 2022, many cities in the Yangtze River Delta region have implemented nucleic acid tests for all inhabitants and even closed down the city for many times. This had undoubtedly interrupted the normal operation of the economy and society to a certain extent, and lead to the distortion of various statistical data. Therefore, we select RES development related indicators and CCCDEI of YRDCC in 2019 to conduct the regression analysis, and the results are shown in Table 2 and Fig 7.

It can be found from Table 3 that AICc is greater than 3, R^2^ is 0.8780 and the adjusted goodness of fit reaches 84.4%. These indicators reveal an overall reliability of the established GWR model, and significant impact of RES development of a city and its neighbors on the DE development in YRDCC.

**Table 3:** GWR model diagnostics results.

**R** ^**2**^	0.8780	**Sigma-Squared**	43.1529
**AdjR** ^**2**^	0.8440	**Sigma-Squared MLE**	33.9724
**AICc**	283.4928	**Effective Degrees of Freedom**	32.2775

In addition, the standardized residual values of all the cities are within the range -2,5 to 2.5, except Suzhou city (as shown in Fig 7). It indicates the development level of DE in YRDCC not only has agglomeration characteristics in geographical space, but also the impact of RES development on DE development is geographically different. These are most likely caused by the factors such as differences in GDP, total retail sales of consumer goods, per capita gross regional product, general budget revenue, household disposable income of urban residents and added value of the tertiary industry between the cities in YRDCC.

When GWR analysis is performed in ArcGIS, an association coefficient report between the dependent variable and each independent variable is also generated. The association coefficient is identified by R^2^, and the result is shown in Table 4. From this table, we can see that the R^2^ values between CCCDEI and the independent indicators are all greater than 0.6, which proves RES development has significant role in promoting DE development from another side. However, the influence of each RES development related indicator on the development of DE is different. Measured by R^2^, general budget revenue has the highest goodness of fit, and total retail sales of consumer goods ranks the second. This could be because general budget revenue directly determines how much to invest in infrastructure related to DE, and the DE industry is an important part of the DE and the total retail sales of consumer goods. Relatively, added value of the tertiary industry has the least goodness of fit. The reason may be that tertiary industry includes many industries, for example, commerce, finance, services, agriculture and culture, and the added value created by DE accounts for only a small part of the added value of the tertiary industry.

**Table 4:** R^2^ between CCCDEI and the independent variables.

Dependent variable	Independent variable	R^2^
**CCCDEI**	*GDP*	0.65
	TPROUDCTVALU	0.60
	AVGGDP	0.69
	BUDGETREV	0.92
	URBANUSEABLE	0.86
	SOCIALSALES	0.91

### 6.2 Analysis of spatial heterogeneity of influencing factors

With the help of ArcGIS for GWR analysis tool, regression analysis coefficients for each influencing factor can also be obtained. Based on the coefficients, the paper analyzes the spatial heterogeneity of the impact of various explanatory variables on the development of the DE in YRDCC, and we find there are significant spatial differences.

#### 6.2.1. Gross domestic product

**Fig 8:**
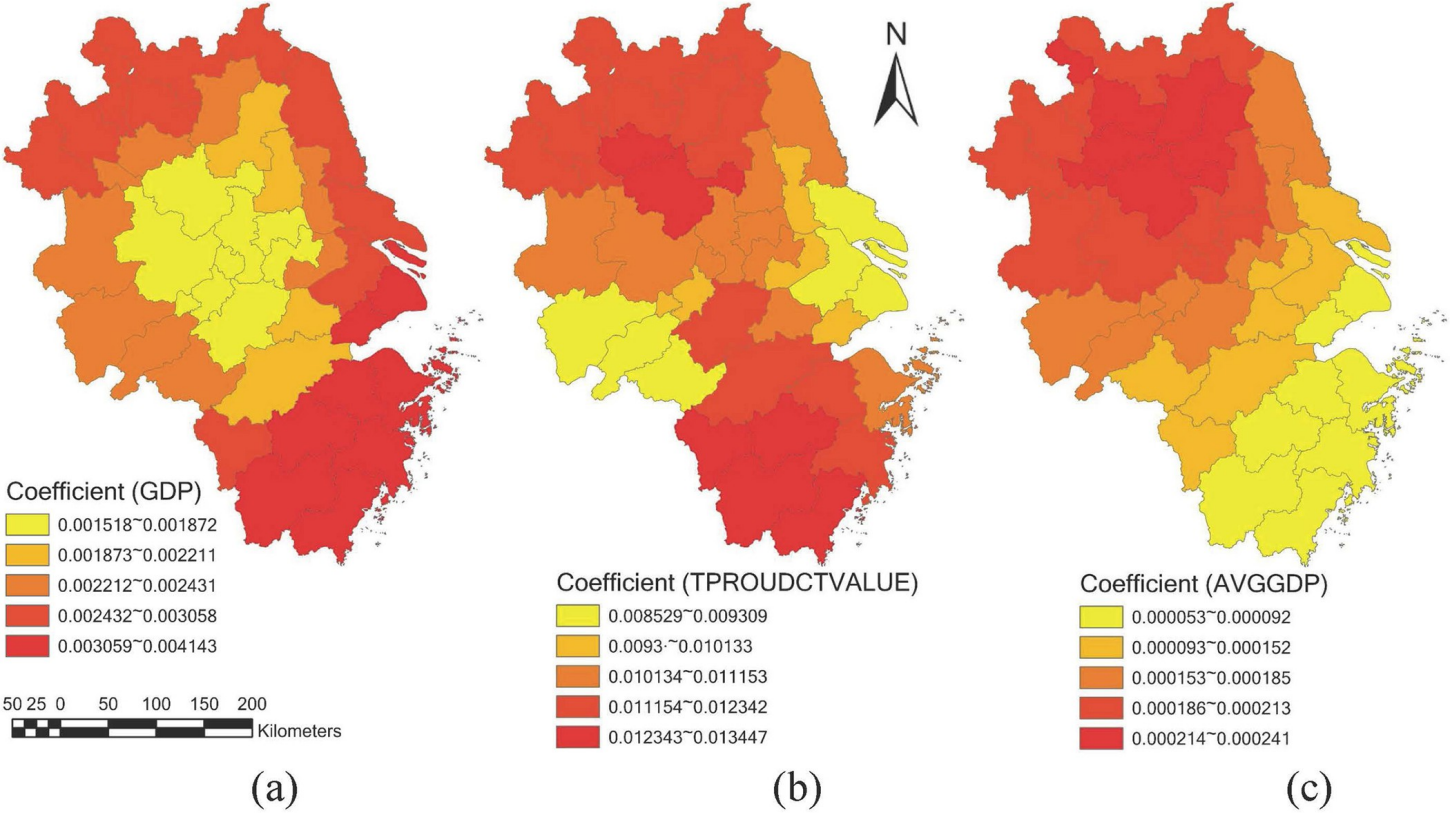
Spatial distribution of coefficients of cities in YRDCC (part 1).

Fig 8(A) indicates a significant positive correlation tendency between DE development and GDP in all the cities of YRDCC and the overall structure shows a decreasing circle structure from the outside to the inside. From the perspective of spatial heterogeneity, high and medium GDP coefficient values are concentrated in the south, north and east regions, and the cities with the highest GDP coefficient are Wenzhou city, Taizhou city, Lishui city, Quzhou city, Jinhua city, etc. While GDP coefficient low values are concentrated in the center area of YRDCC, and the cities with the lowest GDP coefficient values are Chuzhou city, Hefei city, Tongling city, Wuhu city, Xuancheng city, etc. Due to the differences in the economic bases, the increase in GDP of cities in the central region of YRDCC is more conducive to reduce the coefficient difference.

#### 6.2.2. Added value of the tertiary industry

Fig 8(B) reveals an overall positive correlation tendency between DE development and added value of the tertiary industry, and the spatial distribution of added value of the tertiary industry coefficients is somewhat similar to Fig 8(A). Specifically, high and medium coefficient values of added value of the tertiary industry are concentrated in the top and bottom of cities in YRDCC, while low coefficient values are located on the left and right sides. The lowest coefficient cities are Liuan city, Anqing city, Chizhou city, etc. Therefore, reducing the development level of the tertiary industry in YRDCC is one of the effective means to promote the coordinated development of DE in the region.

#### 6.2.3. Per capita gross regional product

Fig 8(C) also shows a significant positive correlation tendency between per capita gross regional product and DE development in all the cities of YRDCC. It implies the higher per capita gross regional product, the higher development level of DE, and this is consistent with existing research findings. On the whole, the spatial distribution of the per capita gross regional product coefficients reveals a gradually increasing trend from south to north. The highest per capita gross regional product coefficient values appear in the northwest region of YRDCC, where the lowest values appear in the southeast. Specifically, the lowest per capita gross regional product coefficient values are located in Lishui city, Wenzhou city, Taizhou city, Jinhua city, etc., and the highest values are located in Suzhou city, Suqian city, Bengbu city, Huaian city, etc. Therefore, vigorously developing regional economy to improve per capita gross regional product level is very important for the development of DE in YRDCC.

#### 6.2.4. General budget revenue

**Fig 9:**
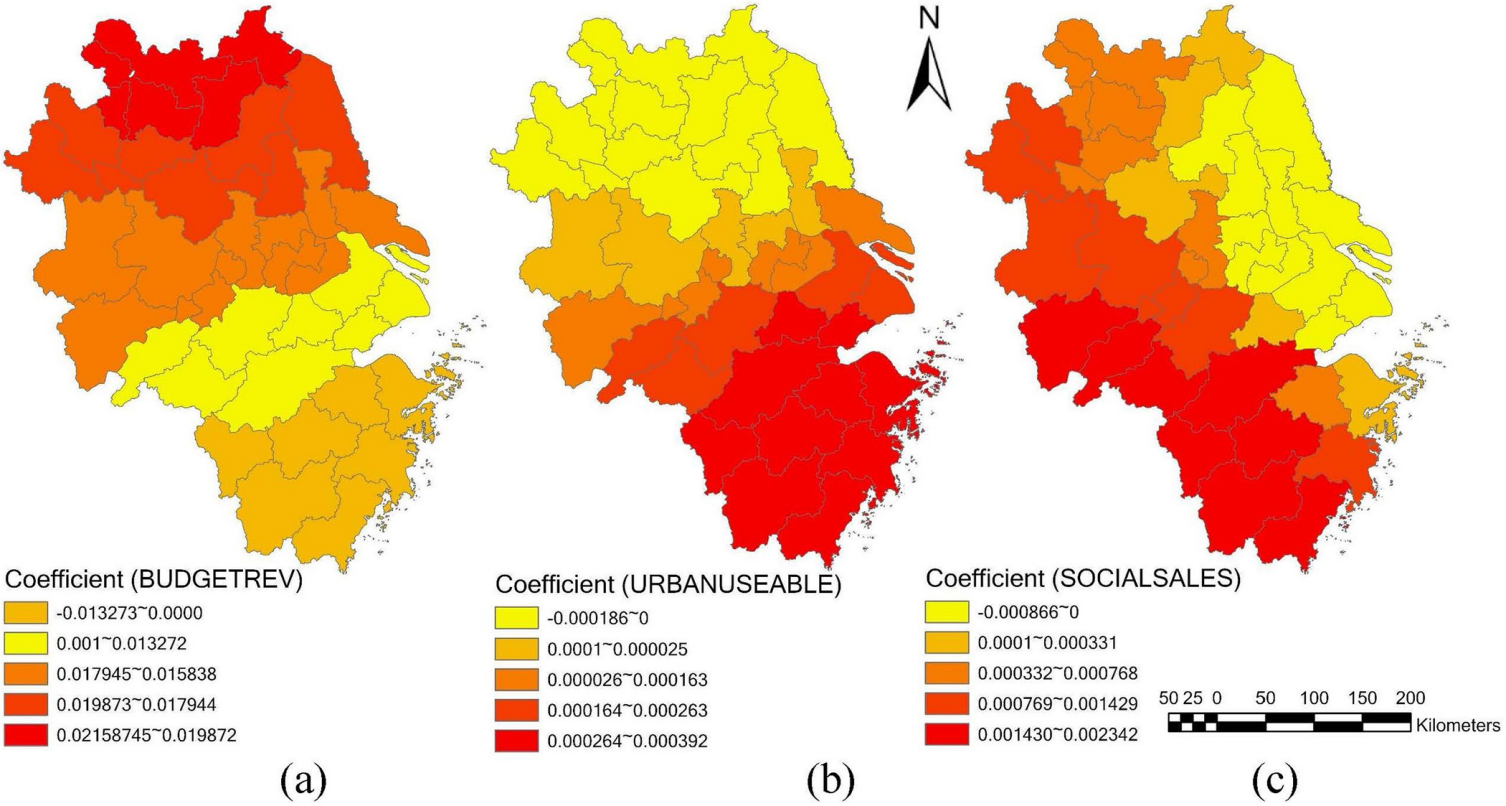
Spatial distribution of coefficients of cities in YRDCC (part 2).

The impact of general budget revenue on the development of DE in YRDCC shows a negative correlation tendency in 19.51% of the cities (as shown in Fig 9(A)). The cities include Quzhou city, Lishui city, Wenzhou city, Taizhou city, etc., which are mainly located in Zhejiang province. Meanwhile, there are 80.49% of cities with positive general budget revenue coefficient values, and the highest values appear in Xuzhou city, Lianyungang city, Suzhou city, Huaibei city, Suqian city, etc. The cities are mainly concentrated in Jiangsu province, and their CCCDEIs are relatively low. Therefore, improving general budget revenue level can also promote the development of DE for cities in YRDCC to a certain extent.

#### 6.2.5. Household disposable income of urban residents

As can be found in Fig 9(B), household disposable income of urban residents’ coefficient values of most cities are positively related to the development of DE in YRDCC. Specifically, 65.86% of the cities shows a positive correlation tendency, while the case for the other 34.14% cities is the opposite. This explains from another perspective that increase the per capita disposable income of urban residents leads to an increase development of DE. The cities with the lowest household disposable income of urban residents’ coefficient values mainly appear in northwest of Jiangsu province and northeast of Anhui province, such as Xuzhou city, Lianyungang city, Huaibei city, Bozhou city, etc. While the cities with the highest values are mainly located in Zhejiang province. These cities include Quzhou city, Lishui city, Taizhou city, Wenzhou city, etc.

#### 6.2.6. Total retail sales of consumer goods

The distribution of total retail sales of consumer goods coefficient values shows a gradually increasing trend from west to east (as shown in Fig 9(C)). Concretely, the impact of total retail sales of consumer goods on DE development in 73.17% of cities indicates a positively tendency. On the whole, the cities with high of total retail sales of consumer goods coefficient values are mainly located in Anqing city, huangshan city, Quzhou city, Lishui city, etc. It implies the development level of DE in these cities is relatively significantly influenced by total retail sales of consumer goods. While the other 26.83% of cities with negative total retail sales of consumer goods coefficient values are generally concentrated in Yancheng city, Taizhou city, Nantong city, Suqian city, etc., which belongs to Jiangsu province.

## 7. Conclusions and future work

Based the existing research findings, the paper investigates the mechanism of RES development promoting the development of DE. To reveal their relationship, we propose a demand-driven regional DE development model. With publicly available dataset, we conduct spatial pattern analysis of DE development in YRDCC, and explore its driving factors. The specific conclusions of this paper are as follows:

First, the spatial imbalance of DE development in YRDCC is very obvious. At provincial scale, DE development level in Shanghai is the highest, while Anhui province is the lowest between 2018 and 2021. At city scale, cities with high DE indices are mostly concentrated in the east of the Yangtze River Delta and coastal areas, while cities with high average annual growth rates are mostly concentrated in the western region.

Second, there are DE development clusters in YRDCC. LSA analysis results indicate that there are two High-High clusters, where are DE well-developed hot places in YDRCC from 2018 to 2021, and two Low-Low clusters, where are DE development cold places at the same time. And GSA analysis results imply there is a less than 1% likelihood that the clustered pattern of DE development in YRDCC could be the result of random chance.

Third, RES development significantly affect DE development in YRDCC. The GWR analysis diagnostics report shows R^2^ values between CCCDEI and the six RES development related indicators are all greater than 0.6. However, the influence of each indicator on the development of DE is geographically different. The quantitative spatial analysis proves RES development is a critical factor that promote the development of DE in YRDCC.

Due to data availability limitation, we collect CCCDEI and RES development related indicators of cities in YRDCC from 2018 to 2021 to conduct the spatial pattern analysis of DE development. The results may not fully represent the diversity of factors influencing the DE at a national or global scale. In the future, we plan to collect more data for consecutive years to carry out spatiotemporal evolution analysis of DE development in YRDCC. In addition, it is worth to collecting other district or county level DE development index to examine its driving factors in finer scale, and make the results more concrete and credible.

## Supporting information

S1 Data (XLS)
